# A park-based group mobility program for older adults with difficulty walking outdoors: a qualitative process evaluation of the Getting Older Adults Outdoors (GO-OUT) randomized controlled trial

**DOI:** 10.1186/s12877-024-05611-z

**Published:** 2025-01-08

**Authors:** Kristina M. Kokorelias, Jacquie Ripat, Ruth Barclay, C. Allyson Jones, Nancy E. Mayo, Theresa Grant, Stephanie Scodras, Kyla Alsbury-Nealy, Charlotte Ryder-Burbidge, Nancy M. Salbach

**Affiliations:** 1 Department of Medicine, Geriatrics, Sinai Health System and University Health Network, Toronto, ON Canada; 2 Rehabilitation Sciences Institute, University of Toronto, Toronto, ON Canada; 3 Department of Occupational Science & Occupational Therapy, University of Toronto, Toronto, ON Canada; 4 Department of Occupational Therapy, University of Manitoba, Winnipeg, MB Canada; 5 Department of Physical Therapy, University of Manitoba, Winnipeg, MB Canada; 6 Department of Physical Therapy, University of Alberta, Edmonton, AB Canada; 7 School of Physical and Occupational Therapy, McGill University, Montreal, QC Canada; 8 Bruyère Research Institute, Ottawa, ON Canada; 9 Department of Physical Therapy, University of Toronto, 160-500 University Avenue, Toronto, ON M5G 1V7 Canada; 10 The KITE Research Institute, Toronto Rehabilitation Institute-University Health Network, Toronto, ON Canada

**Keywords:** Older adults, Outdoor walking, Physical activity, Randomized controlled trial, Task-oriented training, Parks, Community exercise program, Qualitative process evaluation

## Abstract

**Background:**

The Getting Older Adults Outdoors (GO-OUT) randomized controlled trial showed that a workshop and 10-week park-based outdoor walk group (OWG) was superior to the workshop and 10 weekly reminders (WR) with increasing walking capacity, but not outdoor walking activity, health-promoting behavior, or successful aging, among older adults with difficulty walking outdoors. The objective of this planned process evaluation was to explore participants’ perceptions of mechanisms of impact of and contextual factors influencing experiences with the interventions to help explain the observed intervention effects on study outcomes.

**Methods:**

A qualitative descriptive study involving semi-structured interviews conducted at 6-months post-baseline was conducted. A directed content analysis was undertaken.

**Participants:**

We interviewed 27 adults (52% male, 48% female, mean age 76 years) from the OWG (*n* = 13) and WR group (*n* = 14).

**Results:**

We identified two themes including: “Holding Me Accountable to Walk More Frequently”, and “We Walked Farther, With More Ease and Confidence, and We Felt Better”. Participants in both groups described how the OWG and WR programs provided some degree of structure and accountability to others that increased their motivation to walk outdoors. Participants described how the OWG led to improved walking capacity (e.g., increased walking distance) and confidence. Interacting with people during OWG sessions led to a sense of enjoyment, and well-being.

**Conclusions:**

Community programs that incorporate structure, accountability, and opportunities for social interaction, can help improve motivation to increase outdoor walking activity and a sense of belonging for older adults with difficulty walking outdoors. Park-based OWG programs appear to convey additional important benefits related to improved physical function and well-being.

**Trial registration:**

ClinicalTrials.gov NCT03292510 Date of registration: September 25, 2017.

**Supplementary Information:**

The online version contains supplementary material available at 10.1186/s12877-024-05611-z.

## Background

Maintaining the ability to walk outdoors is a priority for older adults [1]. A decrease in walking ability in general has been found to contribute to lower health-related quality of life [2], a loss of independence by limiting ability to move in environments outside the home or residence [3], higher rates of morbidity [4], and increased mortality [5]. More than any other modifiable risk factor, physical activity can help maintain cognitive function [6, 7] and lower cardiovascular disease risk [8–10]. However, 33% of older adults walk outdoors fewer than 3 days a week [11].

Personal and environmental factors strongly influence outdoor walking behaviours. Seminal preventative health models posit that health behaviours, such as walking, depend on an individual’s perceived benefits of behaviours, and barriers to preventive behaviour [12, 13]. Similarly, self-efficacy theory suggests that walking outdoors is influenced by the strength of an individual’s belief in their ability to perform this activity [14, 15]. The natural environment (e.g., weather, geography) and built environment are also strongly related to walking patterns in adults [16–18], as they influence where older adults are able to walk safely [19]. Higher walkability of neighbourhoods is often associated with land use mix (i.e., integration of land used for residential, commercial, and other purposes), street connectivity, aesthetics and safety [20]. Older adults typically engage in walking for active-transportation or walking for leisure and the built environment influences both of these activities [21].

Interventions to improve walking in older adults continue to emerge [22]. Interventions to promote walking that have been delivered by phone or internet have been found to be somewhat effective [23]; however, tailoring interventions to individuals’ needs via one-on-one counseling is more effective to improve walking [23]. The majority of interventions, however, focus on individual behaviours rather than barriers to walking related to the built environment [23]. Given the important role environment has on walking behaviours, interventions geared at improving participation in outdoor walking must address both the individual and environmental factors which influence an older adult’s outdoor community mobility [24, 25]. Few published interventions have used a multilevel approach for encouraging walking outdoors among older adults [22]. Advanced walking interventions among older adults are needed and novel interventions that combine theoretical frameworks and multilevel approaches may improve outcomes [22].

The Getting Older Adults Outdoors (GO-OUT) randomized controlled trial (ClinicalTrials.gov NCT03292510, registered 25/09/2017) aimed to estimate the short- and long-term effects of a 1-day interactive workshop and a 10-week outdoor walk group (OWG) program compared to the workshop and 10 weekly reminders (WR) on increasing outdoor walking activity [24]. Conducted in four urban centers in Canada, the study enrolled 190 older adults living independently who reported difficulty walking outdoors, with evaluations of study outcomes at 0 months, 3 months, 5.5 months, and 12 months. Physical evaluations at 12 months were incomplete due to the COVID-19 pandemic. The interactive workshop involved circulation to eight interactive stations to build skills for outdoor walking. The OWG involved two one-hour sessions per week led by healthcare professionals at local parks, while the WR program involved 10 weekly telephone reminders reinforcing workshop content [24]. Interventions were complex, comprising multiple components and requiring tailoring of activities based on participants’ abilities [26]. The primary outcome was time spent walking outdoors, measured using accelerometry and GPS data, while secondary outcomes included walking capacity, health-promoting behaviors, and successful aging. A protocol for the GO-OUT study has been published [24].

Given the complexity of the interventions, we embedded process evaluations within the trial, which are valuable for understanding implementation of the interventions, contextual influences, and mechanisms of impact [27]. Quantitative findings indicated that the OWG program was not superior to the WR program in increasing outdoor walking activity [28]. The OWG program, however, significantly improved walking capacity by increasing walking self-efficacy from 0 to 3 months compared to the WR program. Our quantitative process evaluation showed that interventions were implemented with high fidelity [29]. The objective of this planned qualitative process evaluation was to explore participants’ perceptions of the mechanisms of impact of and contextual factors influencing experiences with the interventions to help explain the observed intervention effects on study outcomes.

## Methods

### Design

**Fig. 1:**
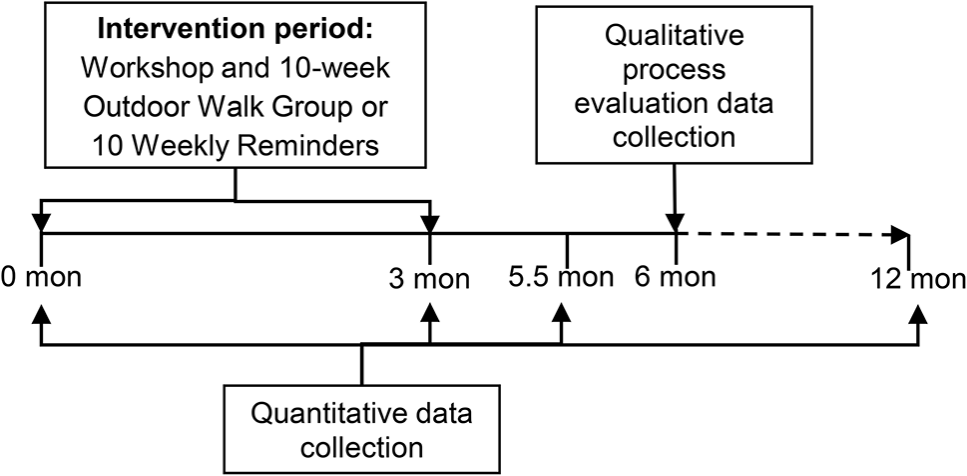
Timeline for data collection in the GO-OUT trial

A qualitative descriptive study [30] was used to address process evaluation objectives as part of the GO-OUT randomized controlled trial (ClinicalTrials.gov NCT03292510). A qualitative descriptive approach was selected as it is useful for yielding practical answers of relevance to policymakers and healthcare practitioners [30, 31] based upon lived experience described from the viewpoint of participants [32]. The qualitative process evaluation was conducted at 6 months post-baseline to optimize participants’ recall of their experiences during and shortly after the intervention period. Figure 1 outlines the timeline for data collection for the qualitative process evaluation in the GO-OUT trial. Details of the trial methodology have been described [24]. The trial was conducted in four urban centres (Edmonton, Winnipeg, Toronto, Montreal) in Canada. Individuals were enrolled in 2018 (cohort 1) and 2019 (cohort 2). Following completion of an interactive workshop, participants were stratified by site and participant type (participation as an individual or with a partner) and randomly assigned to either a 10-week OWG or a 10-week WR program. The research ethics board at each site approved the study procedures. Reporting of this study is in accordance with the Consolidated criteria for reporting qualitative research (COREQ) [33].

### Interventions

#### Workshop

All participants were invited to attend a 5-hour interactive workshop (see Table 1 for workshop components). During the workshop, groups of two to three participants circulated to eight activity stations at which they practiced skills related to the safe walking outdoors (e.g., balance activities, postural awareness), setting goals, using pedometers and walking poles, choosing appropriate footwear and falls prevention [24]. Sites hosted multiple workshops to accommodate participants’ schedules. Fifteen workshops were completed. Three sites completed 4 workshops, and one site completed 3 workshops in May (*n* = 1), June (*n* = 12), or July (*n* = 2).

**Table 1:** Workshop components

Workshop component	Description
Duration	5 h
Format	Interactive workshop with activity stations
Number and Focus of Stations	8 activity stations: 1. Canadian physical activity guidelines for older adults 2. Setting SMART (i.e., specific, measurable, achievable, realistic and timed) goals 3. Pedometer use 4. Nordic pole walking 5. Foot care, footwear, proper walking pattern 6. Falls prevention 7. Monitoring exercise intensity and safety 8. Postural awareness and balance exercises
Group Size	Participants circulated to stations in groups of 2–3
Skills Practiced	Goal setting Using pedometers Using walking poles Choosing appropriate footwear Falls prevention tips Balance activities Postural awareness
Total Number of Workshops	15 workshops
Workshop Distribution across Sites	Site 1: 4 workshops Site 2: 4 workshops Site 3: 4 workshops Site 4: 3 workshops

#### Outdoor walk group (OWG) program

A progressive, task-specific group outdoor walking program was implemented [24, 34]. A health professional (e.g., physical therapist, kinesiologist) led the program with the help of up to two assistants, who were either trained as healthcare professionals or graduate students in health sciences-related programmes, to achieve a participant-to-facilitator ratio of 3-to-1. All assistants received training prior to the start of the program, which included a 90-minute online training session and provision of a comprehensive facilitator guide that reviewed the OWG program, and how to facilitate walking activities, ensure participant safety, and provide support tailored to individual needs. One-hour sessions were scheduled twice each week for 10 weeks. Each session involved a warm-up, practice of walking activities designed to increase capacity for community ambulation (e.g., walking on uneven ground), and a cool-down [24]. Twelve OWGs were run. One site delivered 4 OWGs, two sites delivered 3 OWGs, and one site delivered 2 OWGs. Walk groups ran June to August except for one site that ran three groups August to early October.

#### Weekly reminders (WR) program

Participants in the WR group received a telephone call from the study coordinator, once a week for 10 weeks [24]. During the calls, the study coordinator used a telephone script to provide reminders of information provided at the workshop and discussed with the participant their experiences with outdoor walking [24].

### Participants and sampling

Individuals meeting the following inclusion criteria were considered eligible: (1) age ≥ 65 years; (2) difficulty walking outdoors; (3) living independently in the community; (4) ability to walk at least one block (~ 50 m) continuously with or without a walking aid and without supervision; (5) limited outdoor walking defined as < 75 min/week walking outdoors from May to October (applied to cohort 1 only); (6) willingness to sign a waiver or obtain physician clearance to exercise; (7) mental competency defined by a score of at least 18 out of 22 on the Mini-Mental State Exam telephone; (8) available to participate in the workshop and at least 5 weeks of the OWG program; and (9) able to speak and understand English [24]. Limited outdoor walking was defined as 75 min/week as this represents 50% of the 150 min of moderate-intensity aerobic activity per week recommended in physical activity guidelines for older adults [35], thereby targeting individuals who may benefit from the intervention.

Participants were excluded if they were physically active (i.e., self-reported participation in physical activities for 150 min/week), currently receiving rehabilitation treatment for goals related to walking or at high risk for falls defined using the American Geriatric Society criteria [24, 36].

For the qualitative process evaluation, we purposively sampled participants to obtain a balance of participants across cohorts, study sites, intervention groups, age (i.e., adults 80 and older [37]), and sex (male or female). When recruiting from cohort 2, we purposively recruited individuals who had participated in the study with a partner (i.e., a dyad), and at different frailty levels (i.e., frail, pre-frail, not frail). At study enrolment, all participants indicated on the written consent form that they could be contacted for this qualitative study. At 6 months post-baseline, site coordinators identified participants meeting the sampling criteria who were willing to complete a semi-structured telephone interview. The interviewer obtained verbal informed consent at the start of each interview.

### Data collection

We conducted semi-structured phone interviews. Individuals who participated together (in a dyad) were interviewed together. Prior to each interview, we provided the interviewer with contextual information about each participant related to comorbid conditions, access to a car, walking aid use, attendance in the workshop and attendance in the OWG or the number of weekly reminders received, as applicable.

A PhD-trained, experienced qualitative researcher with a rehabilitation science background (author KMK) conducted all interviews. Semi-structured interviews were selected to encourage the participant to speak freely, while ensuring key topics were discussed [38]. Telephone interviews were selected because participants were geographically dispersed. The interview guide was designed to explore participants’ perceptions of their response to the intervention in the short term and contextual factors influencing their experiences (see Additional file 1 for sample interview guide questions for OWG participants). The GO-OUT conceptual framework [24] was used to guide the interview topics, including effects of intervention components on primary and secondary outcomes, and influence of individual (e.g., disability level, sex) and environmental (e.g., neighbourhood walkability, weather) factors on outdoor walking. Specific prompts in the interview were tailored to participant responses. The interview guide was pilot tested with one participant and then revised by the research team (KMK, NMS, RB, JR). Additional probes were added to the interview guide and refined as data collection progressed. Emerging ideas were discussed among members of the research team (KMK, NMS, RB, JR) to help determine data saturation [39]. The interviewer documented reflexive notes after each interview related to new or recurring comments, emotional responses, or emerging themes.

Our intention was to complete follow up interviews at 12 months post intervention to determine long-term effects; however, this plan was interrupted due to the COVID-19 pandemic. Instead, we focused on the effects of COVID restrictions on outdoor walking and physical activity and aspects of winter walking [40].

### Data analysis

Interviews were digitally recorded, professionally transcribed verbatim, and checked for accuracy by the interviewer by comparing the transcript to the audio file. A directed content analysis [41] guided by the GO-OUT conceptual framework [24] was undertaken. During the first phase of data analysis, the entire research team familiarised themselves with the data from two interviews (one from each intervention group) by listening to audio recordings and/or reading the transcripts and independently generating preliminary codes and taking notes of possible nuances in the data. The research team then met to discuss their preliminary codes including codes based on the GO-OUT conceptual framework [24] related to mechanisms of intervention impact on study outcomes, and contextual factors that could influence intervention mechanisms and outcomes. After the meeting, KMK, in consultation with NMS and RB, familiarized herself with the rest of the transcripts and produced a draft code book (with definitions) to share with the team. The research team met to discuss the codebook and then applied the codebook to one more transcript from the OWG. Another meeting followed to ensure the codebook was not missing any key codes. The codebook was then modified by KMK and NMS and shared with the team for feedback. After the research team finalized the codebook, KMK used the codebook to code the remaining transcripts. NVivo12 software was used to organize the data and facilitate the coding process [42]. Next, authors KMK, NMS, RB and JR reviewed and clustered similar codes to identify emerging categories as they related to the intended and unintended consequences of the interventions, the process by which these outcomes were achieved, and potential modifying influences of individual and environmental factors. Throughout the analysis, particular attention was given to identifying mechanisms through which specific intervention components and contextual factors influencing participants’ walking behaviors and overall experiences. Relationships between categories were explored to identify themes. In situations where participants’ perspectives differed by study site or cohort, these differences were discussed. The same process was followed for data from both cohorts. Once all data were coded, the research team met multiple times to discuss preliminary themes until consensus on final themes was reached.

### Study rigour

Several strategies were used to enhance the credibility of the research findings. Firstly, throughout the process, an audit trail was used with rich, detailed description [43, 44]. KMK maintained the audit trail by documenting when, how and with whom decisions were made at all stages of the research. Memos were also taken before and after each interview and during the transcript accuracy-check. The interdisciplinary team of researchers consulted at all steps of the study process [45]. For example, the entire research team reviewed the data, coded subsets of the transcripts, contributed to code book development, and met regularly to ensure consistency in the definitions and interpretations of the codes into themes. Preliminary results were discussed with the project’s research team to consider alternative interpretations of the data. Throughout the process, discrepancies around emergent ideas, codes, themes, and subthemes were resolved by discussion and reference to the original transcripts by the entire research team to ensure all authors were in agreement. Lastly, we present findings with direct quotations from the participants as exemplary anecdotes that support the findings of the study.

### Sample size

We recruited participants until we had approximately the same number of participants per study site and when a preliminary review of interview transcripts revealed that no new responses were emerging from the data, indicating achievement of thematic saturation [46].

## Results

We conducted 24 interviews with 27 participants. The distribution of participants across sites was: site 1 (*n* = 7, 26%), site 2 (*n* = 6, 22%), site 3 (*n* = 7, 26%) and site 4 (*n* = 7, 26%). Thirteen participants were from the OWG group (48%) and 14 (52%) were from the WR group. Six participants (25%) were part of three dyad interviews. Mean age was 76 years. Fourteen participants were male (52%) and 13 were female (48%). The two most prevalent health conditions were arthritis (19%) and knee replacement (19%). Table 2 presents baseline characteristics of participants by cohort and all participants combined. All participants completed the workshop. Participants in the OWG group attended a median of 85.5% of sessions. Participants in the WR group received all 10 weekly reminders. Interview duration was 36 to 75 min (median 50 min).

**Table 2:** Participant characteristics

Characteristic	Cohort 1 (*n* = 16)	Cohort 2 (*n* = 11)	Pooled (*n* = 27)
	N (%)		
Site			
Site 1	4 (25)	3 (27)	7 (26)
Site 2	3 (19)	3 (27)	6 (22)
Site 3	5 (31)	2 (18)	7 (26)
Site 4	4 (25)	3 (27)	7 (26)
Intervention group			
Outdoor walk group	8 (50)	5 (46)	13 (48)
Weekly reminders	8 (50)	6 (55)	14 (52)
Number in a dyad^†^	0	3	3 (11)
Age in years, Median (P_25,_ P_75_)	75 (70.5, 85.8)	76 (70.2, 80.6)	75 (70.8, 85.7)
Sex			
Female	9 (56)	4 (36)	13 (48)
Male	7 (44)	7 (64)	14 (52)
Level of education			
Secondary or lower	3 (19)	3 (27)	6 (22)
College diploma or taken college or university courses	6 (37)	5 (35)	11 (41)
University degree (undergraduate or graduate)	7 (44)	3 (28)	10 (37)
Marital status			
Single	1 (6)	1 (9)	2 (7)
Married	4 (25)	9 (82)	13 (48)
Widowed	7 (44)	1 (9)	8 (30)
Divorced/separated	4 (25)	0	4 (15)
Retired	15 (94)	9 (82)	24 (89)
Owns a car	14 (88)	10 (90)	24 (89)
Health conditions			
Arthritis	4 (26)	1 (9)	5 (19)
Knee replacement	3 (19)	2 (18)	5 (19)
Parkinson’s disease	2 (13)	1 (9)	3 (11)
Degenerative disk	1 (6)	1 (9)	2 (7)
Mental health disorders	1 (6)	1 (9)	2 (7)
Other^*^	4 (26)	2 (18)	6 (22)
Smoking status			
Never smoked	5 (31)	7 (64)	12 (44)
Ex-smoker	10 (63)	4 (36)	14 (52)
Current smoker	1 (6)	0	1 (4)
Uses a mobility aid	6 (38)	0	6 (22)
Single point cane	3 (50)	0	3 (50)
2-wheeled walker	3 (50)	0	3 (50)
Frailty status			
Not frail	5 (31)	5 (46)	10 (37)
Pre-frail	10 (63)	5 (46)	15 (56)
Frail	1 (6)	1 (9)	2 (7)

Abbreviations: P_25,_ 25th percentile; P_75_, 75th percentile

^*^Included: plantar fasciitis, COPD, stroke, arm paralysis, hypothyroidism

^†^All dyad members were spouses

**Fig. 2:**
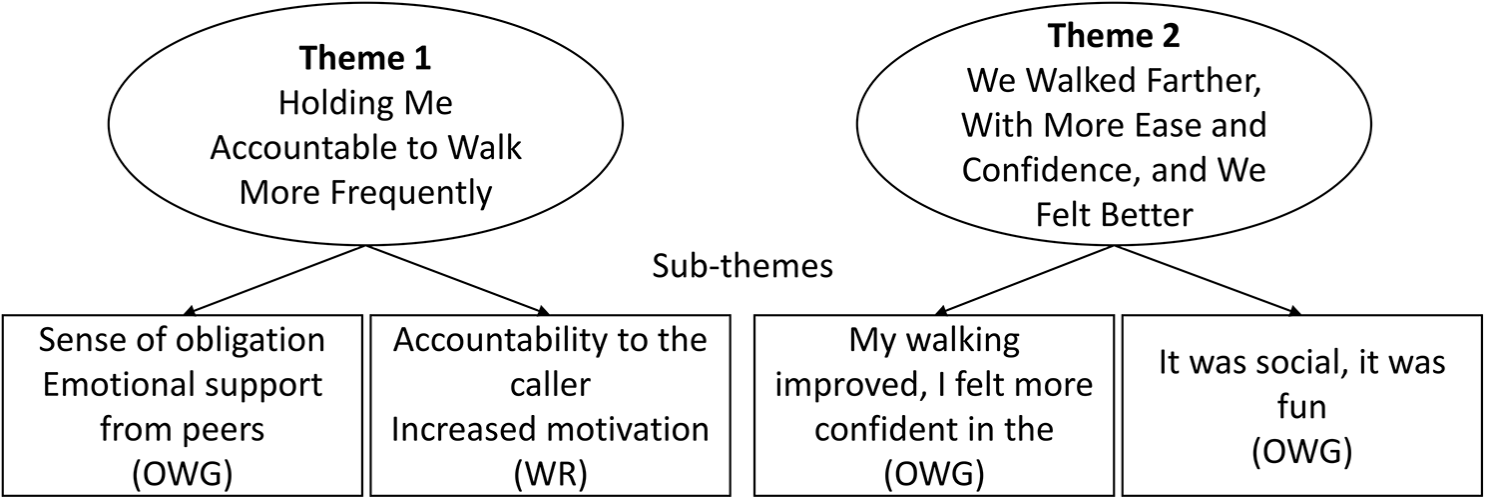
Themes and sub-themes (OWG, outdoor walk group; WR, weekly reminders)

We identified two themes during the analysis. The first theme, “Holding Me Accountable to Walk More Frequently”, described similar experiences of participants with both study interventions. The second theme, “We Walked Farther, With More Ease and Confidence, and We Felt Better”, described OWG participants’ perceptions of the benefits of the OWG intervention on physical and mental health and well-being. In our analysis, we did not note any experiences specific to sex or gender, frailty level, participation as an individual or with a partner, or study site. In general, participants referred to the workshop positively. Figure 2 illustrates the two themes and sub-themes.

### Theme 1: holding me accountable to walk more frequently

Throughout their involvement in the study, almost all participants in both intervention groups reported feeling held accountable to walk more frequently. People in the OWG group noted how the scheduled and group nature of the OWG program increased their motivation to attend OWG sessions and engage with the planned activities. They felt a sense of obligation towards not only the OWG facilitators but also other OWG members. One interviewee noted:

“I think that’s one of the advantages of going to the group session and going out as a group. I think you sort of have an obligation to hold up your end of the group session. Whereas if you were just all by yourself, you could easily talk yourself out of doing it unless you are really, really motivated to do it” (Male, 78 years old, OWG, Edmonton).

Participants in the OWG commented that OWG members shared similar experiences with physical activity and a common goal to increase outdoor walking ability. As participants shared personal struggles during OWG sessions, often the result of mobility challenges, participants provided emotional support through active listening and supportive problem solving. Participants in the OWG also described learning from one another about the benefits of walking that, in turn, increased their motivation to stay physically active.

“And all of us recognized the challenges that we’re all facing. So it was good that we had others that we could talk to. And of course we all were from the same kind of cohort group. So we did have a lot in common as far as growing up like in the ‘60”s when walking wasn’t as popular” (Female, 75 years old, OWG, Winnipeg).

People in the WR group described feeling accountable to the WR caller, as phone conversations reinforced why they should walk, and increased their motivation to walk. Participants frequently referred to the notion of someone else “*holding me accountable*” specifically through active engagement with the WR facilitators.

“Well, I think the personal contact is always good. It keeps you motivated. So, I think that…not a robot call but a real caller, you can ask questions and things too. Which I think is quite valuable if you’re trying to get the incentive to continue walking” (Female, 67 years old, WR, Edmonton).

“Did they annoy me a little bit? Well, maybe. It depends on what I’m doing. But I still want the calls. It was really helpful in reminding me to walk.” (Male, 72 years old, WR, Toronto).

Despite an increased motivation to walk, feelings of accountability did not consistently lead participants in either group to walk outside more often. While individuals in the OWG walked outdoors during scheduled sessions as part of the intervention, they did not describe walking outdoors outside of these sessions or in the follow-up period post-intervention more frequently compared to baseline. Individuals in the WR group did not describe walking outdoors more frequently during and following the intervention phase compared to baseline. Some participants reported being physically active post-intervention (e.g., through gardening or walking in a mall) but not necessarily through outdoor walking.

Participants in the WR group recognized that it was their responsibility to motivate themselves to walk outdoors and make it a habit, but admitted they made excuses for why they did not walk outdoors. One participant stated:

“You know, I sort of like need that commitment to keep me motivated. But you know, when I’m left to do it on my own, I find a million different excuses why I can’t do it.” (Male, 71 years old, WR, Winnipeg).

In alignment with comments made by people in the OWG, some participants in the WR noted that if they had been in the OWG they would have likely participated in walking more during OWG sessions, due to feeling accountable to the group and having to walk at a scheduled time. One participant shared: *“The fact that there would have been a set time*,* a set place…I’m that person that if I make a commitment*,* I’m going to go there and I’m going to do it.”* (Male, 81 years old, WR, Winnipeg).

### Theme 2: we walked farther, with more ease and confidence, and we felt better

Participants in the OWG described how the intervention facilitated improvements in physical and mental health and well-being, benefits that were not noted by participants in the WR group. These findings are summarized in two subthemes.

#### Sub-theme 1: my walking improved, I felt more confident in the OWG

Participants in the OWG group described subtle improvements in walking capacity that included walking greater distances, walking with more ease, walking more quickly, increased strength in their legs, and improved fitness. Participants frequently attributed these improvements to practising a variety of walking tasks during scheduled OWG sessions, which they would not ordinarily try, in the presence of a facilitator (OWG leader) in a park environment. The variety of tasks not only enhanced their practical walking skills but also significantly boosted their confidence to perform new walking skills, as they experienced mastery in challenging situations with the support and guidance of the OWG leader. The facilitator’s presence fostered a safe and supportive atmosphere, enabling participants to take risks and engage more fully in their walking practice. As one participant shared:

“I guess what it did is as we participated in the structure of the study, we noticed a very subtle change in our ability to…with some of our endurance, some of our stamina and being able to walk just a little bit further, a little bit longer, and walking became a bit easier. And we felt better. And we could notice changes in our health and our strength grew.” (Male, 84 years old, OWG, Edmonton).

One participant described pushing themselves physically, as encouraged by the OWG leader:

“[The OWG leader] gave me the confidence I needed. I didn’t feel I had to look down all the time for some reason. It just gave me a balance that I needed. And this is through the park and through the trees that we walked because the ground was uneven. And the physios that were with us, they were very good for that too. (Female, 73 years old, OWG, Montreal).

Many OWG participants described how performing exercises that were repeated during the sessions helped them gain a sense of confidence in their walking abilities and hence helped motivate them to try the various activities. Participants largely attributed this confidence to the practice of walking skills or improvement in walking. Some participants noted how this confidence was sustained even after the intervention ended.

“Well, I think it gave me a lot more confidence to go out and try different things. I mean I’m still a little careful because especially with winter coming, there’s more chance…more risk for falls. But I think that as I’m going into my retirement years, I’m feeling confident that I can develop these skills and keep them up, and not be at risk for falls – at least at my age.” (Female, 65 years old, OWG, Winnipeg).

In contrast, participants in the WR group did not describe any physical or overall wellbeing benefits. Participants in the WR group, however, frequently referred to the knowledge that they had learned about the benefits of walking throughout their experience with the workshop and the weekly calls. As one participant shared: *“I really learned a lot. I think one thing that I think is important for seniors is walking. I think it’s vital to our self-being and our ability to maintain our ability to keep mobile.”* (Male, 80 years old, WR, Toronto).

Almost all participants in the WR group commented on their desire to track steps to share with the weekly caller using the pedometer they had received for personal use and taught how to use during the workshops. WR participants shared that the tracking of steps served as a facilitator of outdoor walking activities. Furthermore, the involvement of the weekly caller in hearing about the walking activity through step counts provided positive enforcement to reduce sedentary lifestyles and adopt more active habits. One participant shared: “*Taking the program was a reminder that*,* you know*,* we can do this on a regular basis. And having the pedometer of course. You know*,* wanting to log the steps.” (*Female, 67 years old, WR, Edmonton).

#### Sub-theme 2: it was social, it was fun

Participants in the OWG talked about improvements in their social network and a sense of belonging as part of a group and well-being. Some participants described feeling *“good talking to the other people”* and that walking as a group made outdoor walking more *“fun”* (Female, 67 years old, OWG, Winnipeg). As one participant quote illustrates: *“Well*,* yeah*,* with other people*,* it was social. It was fun. It wasn’t like we were just meeting the therapist*,* and the therapist saying go walk here. I mean they made it fun.”* (Female, 75 years old, OWG, Toronto). In addition, participants often commented that social interaction resulting from group-based activities helped increase their motivation. Many OWG participants also noted how the OWG leaders and assistants facilitated social interaction. As one participant shared:

“They were so pleasant with everybody. They gave their time to each one of us. And, you know, they were just very interested and listened to any of our complaints or listened to what we said was wrong with us. And they took everything into consideration. They were very good.” (Female, 73 years old, OWG, Montreal).

Participants frequently mentioned that OWG leaders, who were trained health professionals, provided advice that was credible and helped them to be able to participate in the various walking activities. One participant stated *“Well*,* they’re physios. They know what they’re talking about.”* (Female, 73 years old, OWG, Montreal).

Participants in the WR program anticipated that the social aspect of the OWG would have been preferable and would have increased their motivation. Such statements were made despite acknowledgement of the frequent and scheduled calls participants received from trained callers. One participant shared:

“I think I might have enjoyed it even more if I had been in the other group. Where I had an opportunity to walk outdoors socially on a regular basis. Because that would be somewhat…provide additional motivation for me from both those points – of being outdoors and this walking together with others.” (Female, 73 years old, WR, Montreal).

Another participant shared: “*So maybe deep down I was thinking*,* oh*,* I didn’t get to walk with a group. And that’s kind of like what I wanted because it seemed that there’s always more… You have more…for me anyways*,* more motivation.”* (Male, 81 years old, WR, Winnipeg).

Participants in the WR program frequently described the importance of social interaction in their own lives and described an interest in continuing with the study, despite not being placed in the OWG group, due to the frequency of the calls, which helped to provide social support. As one participant shared “*A lot of older folks need to be social […] even if it is just receiving a call from someone in a study helps sometimes.”* (Male, 84 years old, WR, Edmonton).

Despite the majority of WR participants describing a desire to have walked with other individuals, a small number of WR participants described the benefits of being able to walk on their own: *“I don’t want to*,* as I said*,* walk with someone because I don’t…I want to go at my pace” (*Female, 81 years old, WR, Montreal*).*

## Discussion

With global recognition of the use of outdoor walking to promote health [1, 47], our study contributes to the existing literature by highlighting the experiences of older adults with two programs aimed at improving outdoor walking [24]. We identified two themes, specifically: “Holding Me Accountable to Walk More Frequently” and “We Walked Farther, With More Ease and Confidence, and We Felt Better”. While both OWG and WR participants described feeling accountable, the components of the OWG intervention that facilitated participation in outdoor walking activity included group participation, scheduled walking activities that included a variety of walking activities, and credible facilitators. We did not note any differences in experiences as they related to sex or gender, frailty, participation with a partner, or study site. The results can serve as a starting point for further understanding of the processes that can help encourage outdoor walking in older adults. We have identified areas that require further exploration. For example, future studies should incorporate self-reported outcome measures that reflect the desires of participants for socialization.

Theories of health behaviour changes conceptualize that older adults require both internal and external motivation to create habitual exercise habits, such as outdoor walking [48, 49]. The GO-OUT study was theoretically guided by self-efficacy theory [15]. While an existing body of evidence suggests that self-efficacy can predict exercise behaviours [14, 50–52], the theory suggests that this behaviour can only be facilitated when individuals have the appropriate skills to participate in the behaviour [15, 52]. We extend on the existing GO-OUT conceptual framework by highlighting that in addition to needing the appropriate skills, external (e.g., motivation such as feeling accountable to a caller or group) and internal motivation (e.g., motivated to feel physically better, knowledge of the benefits of walking) is required. In our intervention, external motivation was only provided for the duration of the program. Participants in the OWG reported numerous physical and well-being benefits, such as enjoyment, through their participation in an intervention that involved a set schedule for walking activity that involved others. Our study confirms the findings of previous studies that discuss the influence of walking in parks [53–55] and with others [56, 57] on increasing participants’ engagement and motivation in outdoor walking activities. Nonetheless, OWG participants still would have required internal motivation to attend the scheduled sessions. The older adults in this study highlighted the importance of feeling accountable to someone else, resulting in improved external motivation, and described improved knowledge about the benefits of walking that may have influenced internal motivation.

The Cognitive Evaluation Theory [58] and Self-Determination Theory [59] posits that the majority of exercise behaviour is driven by intrinsic (internal) motivation. Such findings can help to explain the quantitative trial results whereby the OWG was associated with greater improvement in walking capacity from 0 to 3 months of the intervention than the WR [28]. As such, future research is warranted to consider how to foster and sustain internal motivation to help encourage outdoor walking behaviours in older adults. Moreover, as social support during exercise can improve self-efficacy [28, 60], an analysis of how older adults’ self-efficacy beliefs differ with increased social support during outdoor walking, is warranted. Other theories, such as the theory of planned behaviours that considers behavioural intentions that are influenced by an individual’s attitude toward a behaviour (i.e., walking outdoors), may help to guide intervention research in this area [61]. Thus, future research is warranted to explore strategies to improve motivational regulation in the context of older adults [62]. For example, an exploration of how knowledge of the benefits of walking may influence walking behaviours long-term should be considered by interventionists. Moreover, cost-effective wearable technologies (e.g., pedometers) and alternative methods for measuring walking ability (e.g., walking logbooks) should be incorporated into future interventions such that motivation is increased and expert knowledge to interpret results is not required.

The finding that participants reported socialization and social support as influential on their walking behaviours and experiences with the interventions supports other recent work [63, 64]. Our study findings underscore the significant role of both social support and the facilitator (OWG leader) in influencing participants’ walking behaviors and experiences with the interventions. Participants reported that socialization and social support were vital components that enhanced their motivation and engagement, aligning with recent qualitative research indicating that reduced social interaction may negatively impact walking activity [65]. The presence of an OWG leader enabled participants to successfully practice and build self-efficacy to perform challenging outdoor mobility tasks. This supports the quantitative findings that showed greater self-efficacy in the OWG compared to the WR group. Other qualitative studies suggest that reduction in socialization may lead to a decrease in walking activity [66] and may help to describe how self-efficacy (as a driver of walking capacity) was greater in OWG than WR group, as found within the quantitative findings. Existing evaluations of randomized walking interventions studies have suggested various potential benefits of improved balance confidence and weight-loss [67, 68], but our study emphasizes the need to explore perceived well-being and social support more deeply. Including validated social support measures in future intervention research may therefore be valuable in trying to measure the effects of walking interventions (e.g., Duke-Social Support Questionnaire [69], Perceived Social Support Questionnaire [70]). In addition, the characteristics of the facilitators (i.e., knowledge, education, attitudes and personalities) were considered important by our study participants. Currently, little is known on how to select and train the best walking intervention facilitators [71]. Our findings may suggest that for an exercise intervention to be effective, it should be sufficiently structured and delivered by healthcare providers who are knowledgeable about the benefits of walking and knowledgeable about behaviour change (e.g., motivation, habit-forming).

### Limitations

The findings must be transferred with caution to other settings and participants which differ. For example, participants were only English-speaking and from four provinces in Canada, thus limiting transferability. None of our participants lived in rural or remote areas. Moreover, strategies to recruit participants for interviews who did not complete the interventions as scheduled (e.g., did not attend most of the group sessions or participate in calls) including asking about reasons for non-participation and demographics (e.g., clinical characteristics) of those who were unable to participate would have provided insights that could inform future interventions to meet diverse needs. Additionally, participants completed interviews by telephone; thus, non-verbal communication such as body language could not be noted and interpreted. The quality of phone interviews has been debated within the literature [72]. Transcripts were not returned to participants to review for accuracy and member-checking was not conducted. While the workshop component of this interview was referred to positively, many participants did not recall specific components of the workshop and the influence they had on their walking habits. Lastly, participants of this study were those who attended the majority of the OWG sessions; thus, we are unable to understand what motivated the people who were less engaged in the study interventions.

## Conclusions

Older adults perceive participating in a structured program as valuable in holding them accountable. Results also indicated that an OWG program with structured activities may impact outdoor walking abilities in terms of distance, stamina, and ease of walking. Structured reminder calls can reinforce the value of walking outdoors for older adults, but these interventions may not necessarily improve outdoor walking capacity. Social support among participants emerged as a key facilitator, as peer connections provided encouragement and reinforced commitment to the walking program. Likewise, the role of the OWG leader emerged as a critical factor in facilitating the practice of challenging outdoor mobility tasks, and their knowledge, support, and facilitation significantly enhanced participants’ experiences. Future research should incorporate outcome measures that reflect participants’ desires and experiences, such as feelings of socialization, enjoyment, and well-being. Additionally, exploring strategies for improving motivational regulation among older adults is essential to achieving long-term and sustainable outcomes in walking interventions.

## Electronic supplementary material

Below is the link to the electronic supplementary material.

Supplementary Material 1: Additional file 1: Sample Interview Guide Questions for Participants in the Outdoor Walk Group

## Data Availability

The datasets generated and/or analysed during the current study are not publicly available as the participant consent forms did not address open public access to the data and due to limitations of the research ethics approval by the health sciences research ethics boards at the University of Toronto, University of Manitoba, University of Alberta and McGill University. Data are available upon request from the corresponding author on reasonable request and subject to research ethics boards review.

## Abbreviations

GO-OUT: Getting Older Adults Outdoors
OWG: Outdoor Walk Group
WR: Weekly Reminders
