# Supplementary material for: A park-based group mobility program for older adults with difficulty walking outdoors: a qualitative process evaluation of the Getting Older Adults Outdoors (GO-OUT) randomized controlled trial
Source: BMC Geriatr. 2025 Jan 8;25:16. doi: 10.1186/s12877-024-05611-z (PMC11707900; doi:10.1186/s12877-024-05611-z)
Supplement: Supplementary file 1 — Supplementary Material 1: Additional file 1: Sample Interview Guide Questions for Participants in the Outdoor Walk Group [file 12877_2024_5611_MOESM1_ESM.docx]

**Additional File 1: Sample Interview Guide Questions** **for Participants in the Outdoor Walk Group**

1. Tell me about your decision to participate in this study?
2. Could you describe your experience with participating in the workshop?
3. Could you please describe whether the workshop helped you in any way and if so, how?
4. What was your experience with the walking group?
5. Could you share any thoughts you have about whether experiences with the walking group would be the same for men versus women; people who use versus do not use a walking aid (eg a walker or cane); people who drive versus do not drive; people who have vs do not have good areas to walk in their neighbourhood. Also influence of weather on walking outdoors.
6. Could you please describe any possible ways the walking group did or did not help you?
7. Could you describe whether you went for walks outside on your own time during the 10 weeks you were in the walking group?
8. Could you please describe your experiences walking outdoors since the outdoor walk group ended 3 months ago?
9. Could you please describe any challenges you experienced participating in the study?
10. Is there anything else you think I should know to understand your experiences with the program or how the program affected your activity level or health?
